# Bronchoalveolar lavage lymphocytosis in hypersensitivity pneumonitis: a retrospective cohort analysis with elimination of incorporation bias

**DOI:** 10.1186/s12890-022-01844-z

**Published:** 2022-02-01

**Authors:** Meghan Hill, Tananchai Petnak, Teng Moua

**Affiliations:** 1 Department of Internal Medicine, Mayo Clinic, Rochester, USA; 2 Division of Pulmonary and Critical Care Medicine, Mayo Clinic, 200 First St. SW, Rochester, MN 55905 USA

**Keywords:** Hypersensitivity pneumonitis, Bronchoalveolar lavage lymphocytosis

## Abstract

**Background:**

Recent studies support the diagnostic role of bronchoalveolar lavage lymphocytosis (BALL) in patients with suspected hypersensitivity pneumonitis (HP). Our study aim was to determine the spectrum of BALL findings with elimination of incorporation bias in non-fibrotic and fibrotic patients and assess correlates of positive BALL cut-off and BALL association with long-term outcomes in those with fibrotic disease (f-HP).

**Methods:**

A single-center retrospective cohort study was pursued of patients undergoing diagnostic bronchoscopy for interstitial lung disease. Strict study enrollment was based on recent ATS/JRS/ALAT diagnostic guidance meeting ‘moderate’ or higher diagnostic confidence. BALL findings were assessed in both fibrotic and non-fibrotic HP patients with regression and survival analysis pursued for correlates of positive BALL cut-off and long-term outcome.

**Results:**

A total of 148 patients (88 fibrotic and 60 non-fibrotic) meeting moderate or higher diagnostic confidence were included. Median BALL in f-HP was 15% compared to 19% in non-fibrotic patients, with only 28% of f-HP meeting diagnostic cut-off (≥ 30%) compared to 41% of non-fibrotic. For f-HP, centrilobular nodules on computed tomography was positively correlated with a diagnostic BALL (OR 4.07; *p* = 0.018) while honeycombing was negatively correlated (OR 6.9 × e^−8^; *p* = 0.001). Higher BALL was also associated with lower all-cause mortality (HR 0.98; *p* = 0.015).

**Conclusion:**

With elimination of incorporation bias, most patients with well-described HP did not meet diagnostic BALL thresholds. Higher BALL was associated with better long-term survival in those with fibrosis, but its diagnostic role may be more additive than characteristic or distinguishing.

## Introduction 

Hypersensitivity pneumonitis (HP) is an immune-mediated interstitial lung disease characterized by inhalational antigen exposure leading to a type IV hypersensitivity response and subsequent parenchymal lung injury [1]. Bronchoalveolar lavage lymphocytosis (BALL) has been recently proposed to support HP diagnosis [2]. A recent diagnostic guideline from the ATS/JRS/ALAT recommends the use of BALL with a lymphocyte count of greater than or equal to 30% as increasing diagnostic confidence [3]. Evidence for this recommendation references prior studies demonstrating comparatively higher BAL lymphocyte counts in HP compared to other interstitial lung disease (ILD) [4–9]. Two recent systematic reviews and meta-analyses report higher BALL in HP though pooled findings suggested less predictive characteristics [4, 10]. The authors highlight several important limitations including the aggregation of older studies with varied diagnostic criteria and incorporation bias, where BALL itself may have been used to make initial diagnoses for study inclusion.

Prognostic significance has also been reported for BALL findings in HP. Higher counts have been associated with improved survival [11, 12] and better response to treatment [13, 14]. However, such findings remain unclear in patients with fibrosis as prior clinical categorizations based on suspected duration of symptoms or antigen exposure (categorized as ‘acute’, ‘subacute’, and ‘chronic’) did not specifically distinguish between fibrotic or non-fibrotic presentations. It is known that fibrosis is associated with greater mortality in HP [15] at the same time also associated with lower BALL counts compared to non-fibrotic patients [16].

The primary aim of our study was to determine the extent and frequency of BALL findings in patients with non-fibrotic and fibrotic HP diagnosed according to recent international guidance, without the inclusion of BALL as a diagnostic criterion. Secondary objectives included evaluating the association of BALL findings with survival as well as assessing clinical correlates of diagnostic BALL findings in those with fibrotic HP, where clinical diagnosis is often more challenging given overlap with other fibrotic ILD and potential increased theoretical risk with invasive studies due to more clinically severe or advanced disease.

## Methods

Institutional review board (IRB) approval was obtained (IRB #20–000,211) prior to study initiation. The experimental protocol was performed in accordance with relevant guidelines and regulations as reviewed by the Mayo Clinic IRB. Subject informed consent was waived by Mayo Clinic IRB given the retrospective design. A computer-assisted search was used to identify individuals 18-years or older with ILD and bronchoscopy as diagnostic and procedural search terms completed at Mayo Clinic (Rochester, MN) from January 1, 2005 to December 31, 2019. Individual cases were reviewed by study members and included if other causes of ILD were excluded and criteria were met for ‘definite’ (> 90%), ‘high’ (80–89%), or ‘moderate’ (70–79%) diagnostic confidence levels for HP, according to the recent 2020 ATS/JRS/ALAT diagnostic guideline [3]. Most importantly, BALL was not included as a diagnostic criterion to specifically avoid incorporation bias, with patients not meeting at least ‘moderate’ diagnostic confidence without BALL excluded for the purposes of this study.

Additional study variables included age, sex, smoker status, date of clinical diagnosis, histopathologic findings, documented exposure history, HP precipitin serology, chest high resolution computed tomography (HRCT) findings, and pulmonary function testing (PFT) (percent predicted total lung capacity (TLC%), forced volume capacity (FVC%), and diffusion capacity for carbon monoxide (DLCO%) according to standard laboratory criteria). Classification of fibrotic versus non-fibrotic HP was defined by the presence of radiologic fibrosis (traction bronchiectasis or bronchiolectasis with surrounding ground glass or reticulation and/or any honeycombing defined by three or more sequential subcentimeter cysts with two or more stacked rows in any lobe) on presenting CT, with individual scans reviewed for additional ground glass opacities (GGO), mosaic attenuation (at sites away from areas of fibrosis), and centrilobular nodules (> 10% involvement of any single or multiple lobes). Scans were also assessed by two study members using criteria described in the 2020 ATS/JRS/ALAT guideline for categorization into ‘typical’, ‘compatible’, or ‘indeterminate’ radiologic presentations, with ‘indeterminate’ findings defined as features pointing away from HP in those with non-fibrotic presentations (absence of GGO, centrilobular nodules or opacities, and mosaic attenuation), and more peripheral than peribronchovascular or airway centric fibrosis and findings typical of other f-ILD. Reports of histopathologic findings from diagnostic surgical or transbronchial forceps or cryobiopsies were also reviewed according to guideline criteria and categorized into ‘typical’, ‘compatible’, or ‘indeterminate’ for both types of fibrotic and non-fibrotic HP.

### Statistical analysis

Statistical analysis was performed using JMP (Version 14.0, Cary NC). Summary statistics were presented as mean and standard deviation or median and 25–75% interquartile range (IQR) for continuous variables and number and percent for categorical variables. Baseline characteristics of patients with fibrotic and non-fibrotic HP were compared using Wilcoxon rank-sum and ${\chi }^{2}$ tests. Presenting case frequencies for various BALL thresholds (10% cut-offs) were collated for fibrotic and non-fibrotic patients. A BALL cut-off ≥ 30% (as proposed by recent international guidance) was used to stratify positive versus negative findings in both subtypes. Univariable logistic regression was performed to determine clinical and radiologic correlates of elevated BALL (≥ 30%) in patients with fibrotic disease.

All patients without reported dates of death in the medical record were reviewed using the United States Social Security Death Index (USSDI). If date of death was not found, date of the last USSDI search minus six months was used as last date of last known alive or follow-up. Survival time was defined in months from the date of initial clinical assessment for ILD to date of death or last known alive, with censoring of live patients. Cox proportional hazards regression was performed in the f-HP group to determine unadjusted and adjusted predictors of all-cause mortality, using a priori selected covariables of age, sex, and presenting FVC% for multivariable analysis. A cubic spline was generated to assess potential linear or nonlinear correlation of calculated hazard ratios (HR) and measured BALL in the f-HP group. Additional survival analysis was not pursued in non-fibrotic HP patients as their comparative survival was generally greater > 10 years. To confirm this, survival was stratified by radiologic fibrosis at presentation using Kaplan–Meier with Log rank analysis, with additional comparison in the fibrotic subgroup stratified by BALL cut-off ≥ 30%. Two-sided *P* values < 0.05 were considered statistically significant.

## Results

**Fig. 1:**
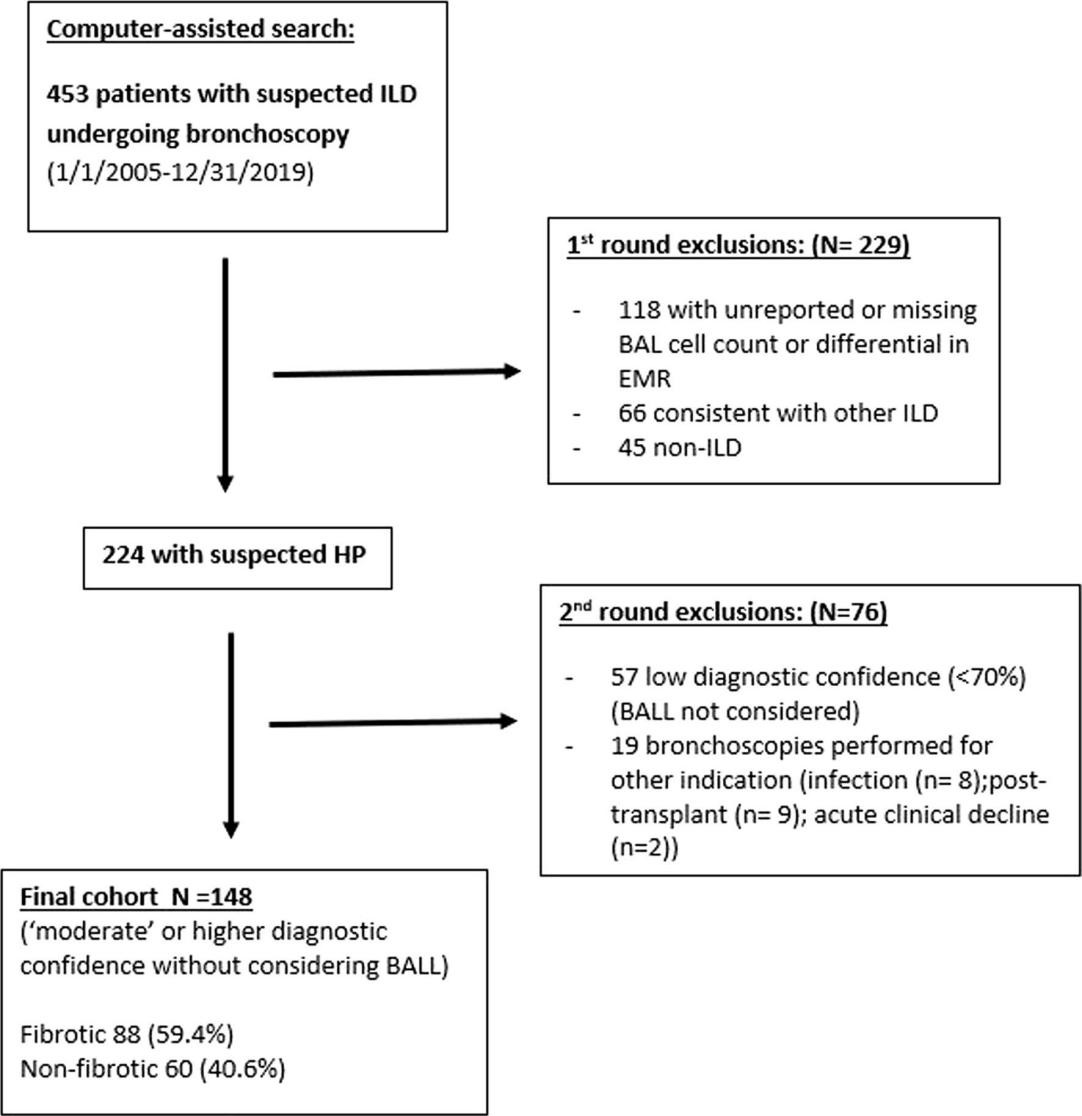
Case selection

A total of 148 (88 fibrotic and 60 non-fibrotic) of 224 screened patients with suspected HP who underwent bronchoscopy were included in the study (case selection presented in Fig. 1). Baseline clinical characteristics and criteria meeting recent consensus guidance for HP diagnosis are presented in Table 1. Fibrotic patients were slightly older (65 vs 60, *P* < 0.0001) with no differences in sex, smoking history, frequency of solicited exposure or serology findings. Fibrotic patients also had lower pulmonary function findings compared to non-fibrotic. Frequency of positive exposure history and precipitin testing was similar between fibrotic and non-fibrotic patients, dominated by avian antigen. Of note, exposure history was solicited and unknown in 43% of fibrotic patients, compared to 23% of those without fibrosis. Specific radiologic findings including GGO (78%) and mosaic attenuation (65%) were found commonly in patients with f-HP, with centrilobular nodules (17%) and honeycombing (17%) occurring less frequently. Histopathology was dominated by consistent or probable HP findings (83% in fibrotic, 95% in non-fibrotic). Based on combinations of clinical findings, ‘moderate’ diagnostic confidence levels were reached in the majority of fibrotic and non-fibrotic pts (62% and 58% respectively), followed by ‘high’ (24% vs 25% respectively) and ‘definite’ (14% and 17% respectively).

**Table 1:** Baseline characteristics and ATS/ALAS/JRS diagnostic findings and diagnostic confidence (N = 148)

Characteristic	Fibrotic (N = 88)	Non–fibrotic (N = 60)	*P* value
Age at dx, years (mean SD (range))	65 (11.6)	60 (13.9)	< 0.0001
Sex, M (%)/F (%)	45 (51)	24 (40)	0.182
Ever smoking history, N (%)	31 (35)	25 (41)	0.429
TLC%, median (IQR 25–75) (N = 105)	69 (60–81)	81 (71–98)	< 0.0001
FVC%, median (IQR 25–75) (N = 130)	61 (52–76)	76 (66–84)	0.0006
DLCO%, median (IQR 25–75) (N = 118)	45 (37–52)	58 (48–72)	< 0.0001
Initial treatment, N (%)			
Corticosteroids Steroid-sparing agent None/observed	66 (75) 8 (9) 14 (16)	50 (83) 1 (2) 8 (13)	0.159
Short-term clinical course (6–12 months), N (%)			0.829
Improved/stabilized Worsened Unknown	70 (80) 8 (9) 10 (11)	48 (80) 4 (7) 8 (13)	
All-cause mortality, N (%)	40 (45)	8 (13)	< 0.0001
Exposure hx, N (%)			0.159
Avian Mold/bacterial Other Multiple Documented unknown	23 (26) 15 (17) 8 (9) 4 (5) 38 (43)	8 (30) 15 (25) 9 (15) 4 (7) 14 (23)	
Precipitin testing, N (%)			0.391
Positive avian Positive mold/bacterial Positive multiple Negative Not obtained	22 (25) 6 (7) 6 (7) 42 (48) 12 (14)	9 (15) 3 (5) 3 (5) 31 (52) 14 (23)	
Individual radiologic* findings			
GGO, N (%)	69 (78)	50 (83)	–
Mosaic attenuation, N (%)	58 (65)	36 (60)	–
Centrilobular nodules, N (%)	15 (17)	18 (30)	–
Reticulation/traction bronchiectasis, N (%)	82 (96)	–	–
Honeycombing, N (%)	15 (17)	–	–
Overall CT pattern for HP, ** N (%)			0.477
Typical Compatible Indeterminate	52 (59) 22 (25) 14 (16)	32 (53) 10 (17) 18 (30)	
Histopathology findings**, (N = 133)	(N = 72)	(N = 58)	0.125
Consistent, N (%) Probable, N (%) Indeterminate, N (%)	42 (51) 23 (32) 10 (14)	40 (69) 15 (26) 3 (5)	
Overall ATS/JRS/ALAT Diagnostic Confidence Level**			0.844
Moderate, N (%) High, N (%) Definite, N (%)	55 (62) 21 (24) 12 (14)	35 (58) 15 (25) 15 (17)	

*Comparison not made given distinguishing radiologic findings between fibrotic and non-fibrotic subgroups

**Statistical comparison of frequencies or distribution of findings for each subtype, not the findings themselves (as they differ by subtype accordingly)

BAL percent differential and lymphocyte findings as stratified by fibrotic and non-fibrotic subgroups are presented in Table 2. Median BALL was 15% (IQR 3–31) in fibrotic patients compared to 19% (IQR 10–47) in non-fibrotic (*P* = 0.005). Among patients with fibrotic disease, the largest proportion were those with BALL less than 10% (42%). Only 28% of patients with f-HP had BALL ≥ 30% compared to 41% of patients with non-fibrotic disease (*P* = 0.09).

**Table 2:** Bronchoalveolar percent differential and lymphocytosis distribution

Characteristic	Fibrotic (N = 88)	Non–fibrotic (N = 60)	*P* value
Lymphocyte (%), mean, SD (median, (IQR 25–75))	19, 18 (15 (3–31))	29, 23 (19 (10–47))	0.005
Neutrophil (%), mean, SD (median, (IQR 25–75))	23, 22 (15 (7–33))	19, 19 (15 (4–28))	0.289
Macrophage (%), mean, SD (median, (IQR 25–75))	56, 24 (57 (39–79))	51, 24 (53 (26–71))	0.245
BAL lymphocytosis, N (%)			
< 10%, N (%)	37 (42)	13 (22)	–
10–19%, N (%)	19 (22)	20 (33)	–
20–29%, N (%)	7 (8)	2 (3)	–
30–39%, N (%)	8 (9)	5 (8)	–
40%–49%, N (%)	6 (7)	7 (12)	–
50–59%, N (%)	7 (8)	3 (5)	–
> 60%, N (%)	4 (5)	10 (17)	–
Total ≥ 30%, N (%)	25 (28)	25 (41)	0.09

Table 3 represents univariable correlates of diagnostic BALL (≥ 30%) in patients with f-HP, pursued specifically given recent interest in the use of BALL to differentiate or support f-HP diagnosis but with theoretically greater procedural risk. Presenting clinical findings including demographics (age, sex, and smoking history), exposure history and type, pulmonary function, and serology findings did not appear to correlate with positive BALL. Presence of any centrilobular nodules on CT was positively correlated with diagnostic BALL (OR 4.07 (1.28–12.93); *P* = 0.018), while any honeycombing was negatively correlating (OR 6.9 × e^−8^ (0–1.07e^−19^); *P* = 0.001).

**Table 3:** Univariable clinical predictors of BALL ≥ 30% in patients with fibrotic hypersensitivity pneumonitis (N = 88)

Characteristic	OR (95% CI)	*P* value
Age*	0.98 (0.94–1.02)	0.392
Male sex	0.74 (0.29–1.91)	0.542
Ever smoker	0.52 (0.18–1.49)	0.210
TLC%*	1.01 (0.97–1.05)	0.467
FVC%*	1.00 (0.98–1.03)	0.763
DLCO%*	1.01 (0.97–1.04)	0.524
Any positive HP serology, (excluding untested, N = 12)	1.01 (0.36–2.83)	0.978
Any identifiable exposure history (excluding those with unknown exposures, N = 38)	1.38 (0.52–3.61)	0.508
Avian	1.58 (0.47–5.65)	0.457
Mold/bacterial	0.96 (0.25–3.38)	0.948
GGO	0.76 (0.26–2.45)	0.638
Mosaic attenuation	1.04 (0.39–2.93)	0.927
Centrilobular nodules	4.07 (1.28–12.93)	0.018
Honeycombing	6.9 × e^−8^ (0–1.07e^−19^)	0.001

^*^Unit risk ratios

**Fig. 2:**
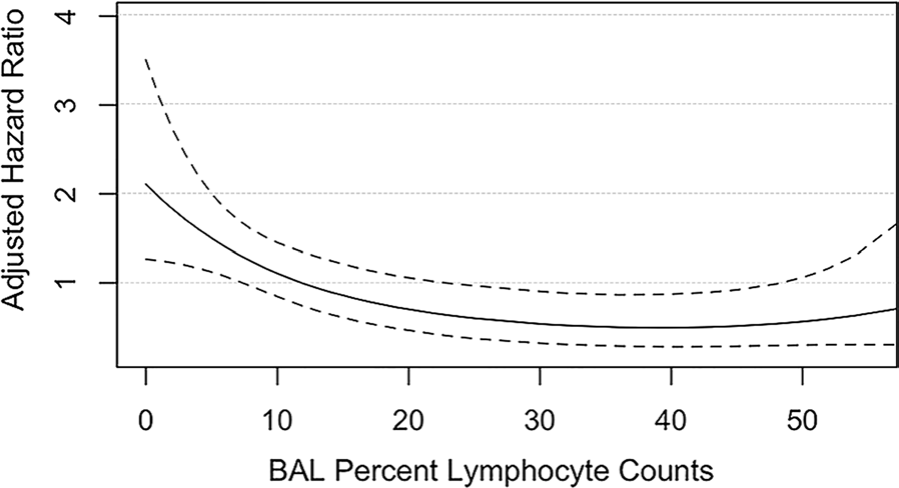
A cubic spline demonstrating non-linear relationship between bronchoalveolar lavage lymphocytosis and predicted hazard ratio for all-cause mortality, with 95% confident interval (dash line), in patients with fibrotic hypersensitivity pneumonitis

Univariable and multivariable Cox regression predictors of all-cause mortality for fibrotic patients are presented in Table 4. Higher BALL as a continuous variable was associated with adjusted and unadjusted lower all-cause mortality (adjusted HR 0.98 (0.96–0.99); *P* = 0.015). A cubic spline representing the relationship between BALL and predicted adjusted hazard ratio in patients with f-HP is presented in Fig. 2. Increased HR was observed with lower BALL counts. A categorical BALL cut-off ≥ 30% was associated with decreased risk of death on univariable analysis (HR 0.431 (0.19–0.95); *P* = 0.027) but not after adjustment for a priori covariables (HR 0.47 (0.21–1.08); *P* = 0.058). The other only independent predictor of mortality was radiologic honeycombing (HR 2.64 (1.15–6.06); *P* = 0.022).

**Table 4:** Univariable and multivariable Cox proportional hazards regression analysis of all-cause mortality predictors in patients with fibrotic hypersensitivity pneumonitis (N = 88)

Characteristic	HR (95% CI)	*P* value	HR¶ (95% CI)	*P* value
Age*	1.07 (1.02–1.11)	0.0003	1.09 (1.04–1.14)	0.0002
Male sex	1.42 (0.75–2.71)	0.276	–	–
Ever smoker	1.60 (0.83–3.08)	0.165	–	–
Any identifiable exposure history	0.68 (0.35–1.29)	0.241		
TLC%*	0.98 (0.96–1.01)	0.147	–	–
FVC%*	0.99 (0.98–1.02)	0.786	–	–
DLCO%*				
Any positive HP serology (excluding those not done, N = 12)	1.34 (0.68–2.67)	0.398	–	–
BALL %*	0.97 (0.95–0.99)	0.004	0.98 (0.96–0.99)	0.015
BAL lymphocytosis ≥ 30%	0.431 (0.19–0.95)	0.027	0.47 (0.21–1.08)	0.058
GGO	0.75 (0.37–1.56)	0.466	–	–
Mosaic attenuation	0.67 (0.36–1.29)	0.238	–	–
Centrilobular nodules	0.27 (0.08–0.89)	0.011	0.31 (0.09–1.02)	0.054
Honeycombing	2.65 (1.23–5.70)	0.012	2.64 (1.15–6.06)	0.022

^¶^Adjusted for a priori covariables of age, sex, and FVC%

^*^Unit risk ratios

**Fig. 3:**
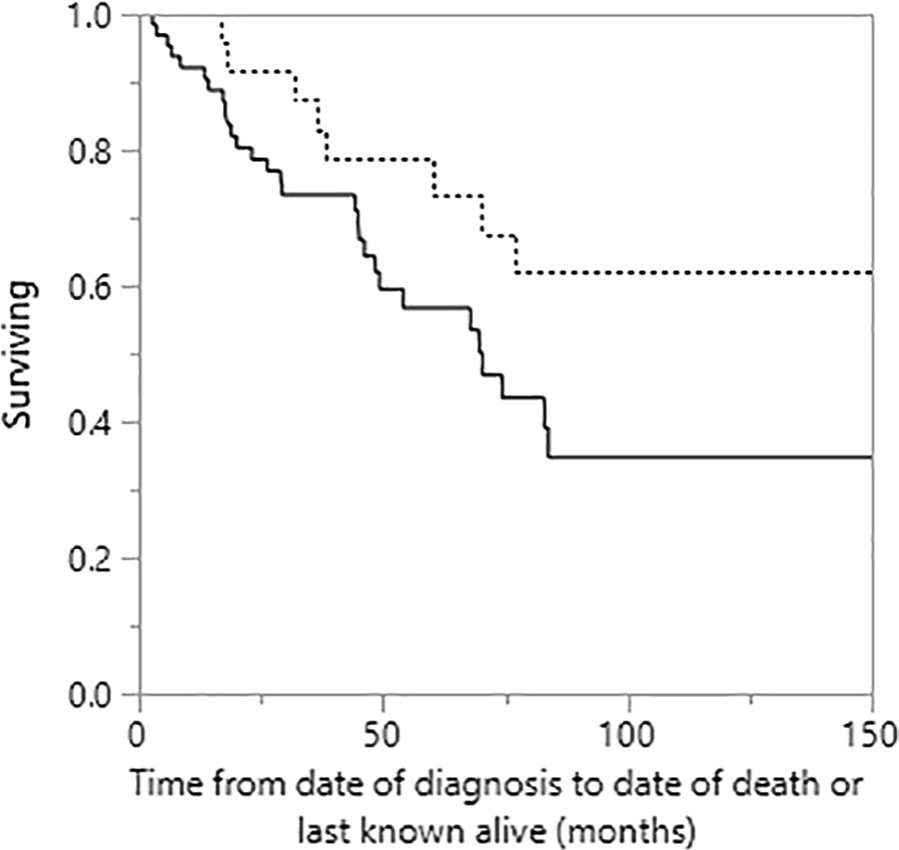
Kaplan–Meier comparison of survival between f-HP with BAL lymphocytosis ≥ 30% versus < 30% at presentation (Log rank = 0.034; BAL lymphocytosis ≥ 30% median survival: 262 months, BAL lymphocytosis < 30% median survival: > 70 months)

**Fig. 4:**
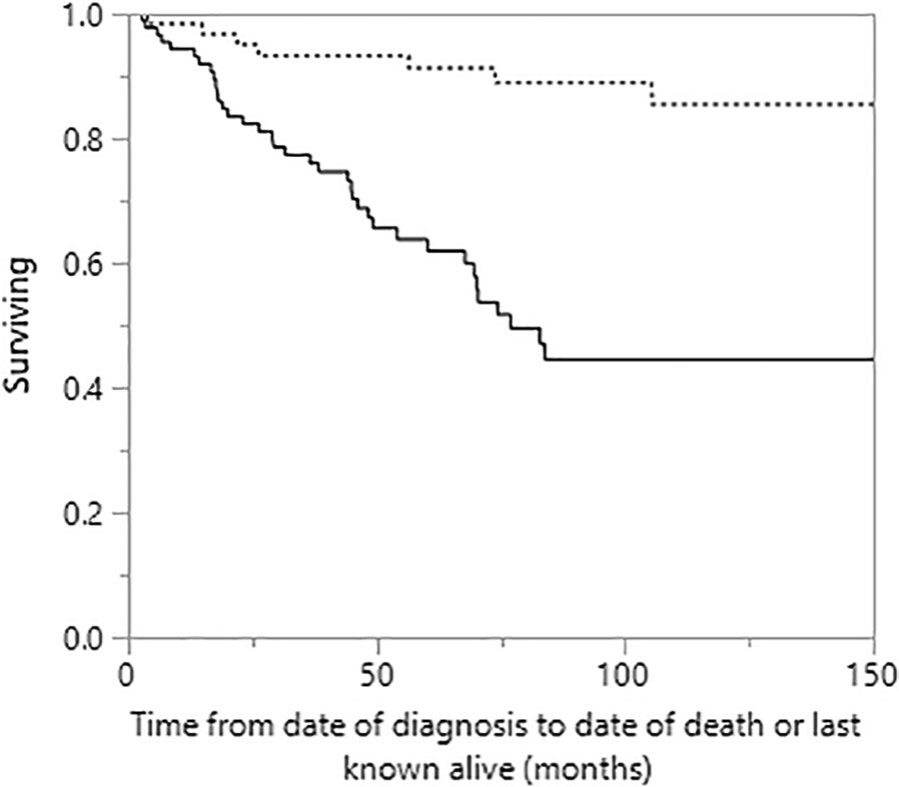
Kaplan–Meier comparison of survival between fibrotic and non-fibrotic hypersensitivity pneumonitis (Log rank =  < 0.0001; f-HP median survival (bold): 77 months, non-fibrotic HP median survival (dashed): > 200 months)

Kaplan–Meier comparison of survival in f-HP as stratified by diagnostic BALL cut-off is presented in Fig. 3. Patients with BALL ≥ 30% at presentation had better survival (Log rank 0.034, median survival 262 months versus 79 months). Kaplan–Meier survival between fibrotic and non-fibrotic HP is presented in Fig. 4. Long-term outcome was worse for fibrotic patients with a median survival of 77 months compared to greater than 200 months (median not reached) in non-fibrotic (Log rank < 0.0001).

## Discussion

Prior studies assessing the diagnostic and prognostic utility of BALL in fibrotic and non-fibrotic HP may be limited by varied diagnostic criteria and incorporation bias, hindering broad application to current practice. Our findings are derived from a cohort of well-characterized HP patients meeting at least ‘moderate’ or higher diagnostic confidence levels according to recent 2020 ATS/JRS/ALAT diagnostic guidance, without the addition of BALL as a diagnostic criterion [3]. Using this approach, we found lower frequencies of diagnostic BALL in both fibrotic and non-fibrotic subtypes and higher comparative BALL counts in non-fibrotic versus fibrotic patients. There was also correlation of BALL with long-term survival in those presenting with fibrosis.

Our study confirms BALL counts are lower in fibrotic compared to non-fibrotic HP patients (median BALL 15% vs 19% respectively, *P* = 0.005). Higher BALL counts and increased frequency of positive cut-offs have been previously reported in non-fibrotic patients. Adams et al. reported median BALL of 46% and 19% respectively in 17 non-fibrotic and 60 fibrotic patients undergoing diagnostic bronchoscopy [5]. Takei et al. applied a recent Delphi consensus statement for f-HP diagnosis to consecutively presenting ILD patients (using a BALL cut-off > 40%) and found only 19% of MDD-diagnosed fibrotic patients had diagnostic BALL findings [17]. Frequency of diagnostic BALL was higher at 40% in one study using a lower diagnostic cut-off of 20% [16]. Median BALL was only 16% in another study of 160 chronic HP patients (85% with reported fibrosis) diagnosed according to prior criteria proposed by Schuyler and colleagues [11, 18]. Caillaud and colleagues reported BALL findings in 139 HP patients from five French centers categorized according to presentations of acute, subacute, or chronic disease classification [6]. BALL findings in this study were much higher with a mean of 42% in those classified as chronic. We again eliminated incorporation bias in our study by reviewing only patients meeting ‘moderate’ or higher diagnostic confidence levels without the contribution of BALL. With that approach, only 28% of fibrotic and 41% of non-fibrotic patients met current consensus criteria for diagnostic BALL. More so, among patients with fibrotic disease, the largest proportion were those with BALL counts less than 10% (42%), as might be observed in healthy non-smokers [19].

Depending on diagnostic cut-off and study-specific criteria for inclusion, reported frequencies of diagnostic BALL varied significantly but occurred in less than 50% of cases in most studies, particularly when BALL was being assessed for diagnostic utility. This lower frequency suggests likely overlap with other ILD, as demonstrated by the findings of two recent meta-analyses [4, 10]. Both found lower range sensitivity and specificity for BALL as a diagnostic predictor (depending on cut-off 20% vs 30%), with expected inverse relationship between sensitivity and specificity (higher specificity at the cost of lower sensitivity). This was particularly true among fibrotic patients where the AUC for differentiating f-HP from IPF or sarcoid was reported as poor at 0.54 and 0.44 respectively in one meta-analysis [4]. Lower sensitivity and specificity with poor predictive characteristics for distinguishing f-HP from other fibrotic ILD, suggests BALL may be more additive in conjunction with other diagnostic elements than relied upon on its own for diagnostic confidence given its lower sensitivity.

As such, in patients with suspected f-HP and often more clinically severe disease, predicting procedural yield may be relevant to minimize bronchoscopy-related risk or complications. We assessed pre-procedural parameters in those with fibrosis and found no presenting demographic, exposure type, pulmonary function, or positive precipitin serology characteristic correlated with a greater likelihood of diagnostic BALL. Only radiologic findings of centrilobular nodules and honeycombing appeared to support higher and lower likelihoods of diagnostic findings respectively. A similar correlation of BALL findings (using a 20% cut-off) with radiologic honeycombing was reported by De Sadeleer and colleagues [16]. Honeycombing was found in 50.9% of those with lower BALL compared to only 13.9% of those with higher BALL. Considering the higher rate of honeycombing in their study compared to ours (36% vs 17%), no patient with honeycombing reached diagnostic BALL in our study.

BALL has also been associated with long-term outcomes [13, 14]. A study of 160 chronic HP patients found a positive association of higher BALL with improved survival [11]. De Sadeleer and colleagues reported improved survival in fibrotic patients stratified by BALL cut-off > or < 20%, with stratification by honeycombing also having similarly poor survival when present. BALL was inversely correlated with honeycombing in their study, with the latter on adjusted Cox regression remaining predictive of poorer outcome while BALL was not. We reviewed univariable and multivariable predictors of all-cause mortality in our fibrotic cohort and found higher BALL at presentation to be independently predictive of survival. As BALL was also inversely colinear with honeycombing in our study, we did not adjust for honeycombing in the Cox regression model, noting again none with honeycombing had a diagnostic BALL in our cohort.

Our study has several limitations. First, its retrospective design can only assume correlation but not causation. We also did not specifically compare BALL findings in HP to other ILD subtypes, focusing on reporting BALL in carefully selected patients meeting updated and strict diagnostic guidance, categorized according to levels of diagnostic confidence. Our primary goal was to apply this recent diagnostic approach with elimination of incorporation bias to better obtain a sense of relevant BALL findings in otherwise diagnosable disease. This methodologic approach may select for patients whose diagnoses were already likely to have been made without BALL and therefore cannot explore the role of BALL in increasing diagnostic confidence in those whose presenting data are inconclusive or missing. However, based on our findings, bronchoscopy appears to be unjustified as positive BALL results in otherwise diagnosable disease also appears to be low. Most patients in our study also underwent biopsy to achieve inclusion criteria of ‘moderate’ or higher diagnostic confidence levels, suggesting more aggressive assessment was still pursued despite available BALL findings, which may not be typical of real-world practices (as reflected for example in the study by Adams et al. [5]). This obtaining of additional biopsy is perhaps the result of lower overall BALL findings in our cohort or clinically unhelpful elevated findings without other supportive criteria.

## Conclusion

In a cohort of well-described HP patients meeting ‘moderate’ or greater diagnostic confidence levels without incorporation bias, most fibrotic and non-fibrotic HP patients did not meet a diagnostic BALL threshold of ≥ 30% despite presenting with other typical clinical, radiologic, or histopathologic findings. Only radiologic findings of centrilobular nodules and honeycombing correlated with greater and lower likelihood of diagnostic BALL in those with fibrosis, with higher BALL findings being independently associated with better long-term survival. Based on our findings, BALL appears to be prognostic, but further research is needed to define its diagnostic role, particularly in those with fibrotic presentations.

## Data Availability

The datasets generated and/or analyzed during the current study are not publicly available due to institutional review board limitation but are available from the corresponding author on reasonable request.

## Abbreviations

BAL: Bronchoalveolar Lavage
BALL: Bronchoalveolar Lavage Lymphocytosis
HP: Hypersensitivity Pneumonitis
f-HP: Fibrotic Hypersensitivity Pneumonitis
ILD: Interstitial Lung Disease
IRB: Institutional Review Board
HR: Hazard Ratio
HRCT: High Resolution Computed Tomography
PFT: Pulmonary Function Testing
FVC: Functional Vital Capacity
GGO: Ground Glass Opacities
TLC: Total Lung Capacity
DLCO: Diffusion Lung Capacity
USSDI: United States Social Security Death Index
ATS: American Thoracic Society
JRS: Japanese Respiratory Society
ALAT: The Latin American Thoracic Association
IQR: Interquartile Range
OR: Odds Ratio
